# Mr. Suttleffe's Medical and Surgical Cases, the Result of 38 Years' Practice

**Published:** 1825-01-01

**Authors:** 


*Medical and Surgical Cases; selected during a Practice of Thirty-eight Years.* By Edward Sutleffe, Queen Street, London. 8vo. pp. 628. August 1824.

This work incontestibly proves, that fun and physic are not incompatible substances, especially where the *latter* is dispensed—not swallowed.

Mr. Sutleffe relates his *experience*, not in the whining cant of a methodist, with long face, down-cast eyes, and twirling thumbs—but in a fine original vein of serio-comic earnestness and jocular piety, of which we shall adduce some exquisite specimens anon. Of anecdote Mr. Sutleffe has been a rare collector—nor are wit, humour, (or at least desperate attempts at them) and satire scattered with sparing hand through this curious volume. He has many a sly fling at the M. D's and more than once hints that they ought to go to school to the old general practitioners, to gain medical information. Whether the result of our author's thirty-eight years' experience, now published, shall impress the new society of physicians with a conviction of this necessity we know not—but one thing is certain, that Mr. Sutleffe will be canonized as a medical SAINT among the godly of this metropolis in consequence of the present performance. His motto is—

"In every work regard the writer's *end.*"

But for our own parts we do not see clearly what the public has to do with either the *end* or the *beginning* of a writer. It is with his book they have business—and Mr. Sutleffe's very appropriately *ends* with the "application of leeches to the anus"—having begun, somewhat ominously, with the subject of *mental derangement*, and the great efficacy of "ground ivy" in that wide spreading complaint. So great indeed was our author's success with this remedy, in the beginning of his practice, that he had thoughts of becoming a mad doctor—"until a few unsuccessful cases checked his confidence."—However, he "had an urgent *message* to attend the Duchess of Chandos," who was non compos in consequence of the death of the Duke—but whether he went, or what success he had with her Grace, is not stated. At this period, "our late beloved King George III, *of scripturally pious and blessed memory*, laboured under a paroxysm," and our author, "so sanguine and patriotic were his feelings," waited on Dr. Reynolds to propose the "ground ivy," but here we are left in the dark respecting the result.

While relating an unsuccessful case, Mr. Sutleffe, incidentally discloses his ideas respecting the pathology of mania, which, according to his *post mortem* researches, consists in an enlarged state of the cerebral vessels, *producing* "a constant impetus of blood, quite incompatible with correct ratiocination." Whether Mr. Sutleffe's memory begins to fail, we know not; but after relating several cases of insanity where ground ivy completely failed, he adds this paragraph:—"I cannot call to mind an individual case of mania where the glecoma hederacea has had a full trial, without effect, and eventually recovery." P. 8.

We shall be able to touch on but a very few of the beauties of this most wonderful volume. We must, however, treat our distant readers with a few specimens.

1. In a case of puerperal fever, Dr. John Sims and our author were completely baffled, and had only the mortification "to witness deeper advances towards despondency;" in the midst of which "the scene partook not of the house of mourning, but might rather be thought a little epitome of the abode of bliss." Being pressed for a prognosis, our author stated that, "in reference to the laws of the obstetric art," the patient must die; but as Dr. Sims and himself had now turned over the case to God, it was possible she might recover. It does not appear that the Deity undertook this case of puerperal fever, for the lady died, and, says our author, "the *mystery* was developed immediately on her decease." What this *mystery* was, we are not told—unless the reader can glean it from the following sublime and "scripturally pious" passage.

"Fraught with instruction in its progressive stages, this case may be said to have been singularly honoured in its termination; for the children of the deceased came forth with one consent to join themselves in a perpetual covenant to the Lord and his church, where they now shine as polished pillars in the temple of truth and grace.

Saints, at your heavenly Father's word,  
Give up your comforts to the Lord;  
He shall restore what you resign,  
Or grant you blessings more divine." 19.

2. The next scene which our author describes was not quite so celestial. Mr. Sutleffe and Dr. R—— were attending a patient in Holborn, "where the healing art proved abortive." On day, while they were entering the doctor's carriage, they indulged rather freely in a laugh—most likely at one of Mr. Sutleffe's anecdotes; but their merriment was somewhat interrupted by the following exclamation from the sick house—"*See how these doctors are humbugging us!*" This brought the two Medicos as quickly to their senses as if each had swallowed a glass of the "juice of ground ivy." The doctor quailed for his next day's fee—and all hope of a bonus on Mr Sutleffe's bill vanished into air—thin air.

3. *After pains* are to Mr. Sutleffe quite inexplicable, "no obstetric professor has hitherto been able to assign a consistent cause for them." "We should think certainly," says our very intelligent author, "that the non-existence of nerves in the funis would preclude the possibility of transmitting impressions to the fœtus in utero, and there are those who absolutely deny altogether that this occurs. But such persons cannot have largely mingled with the social circle." What, in the name of Heaven, has the existence or non-existence of nerves in the funis to do with after-pains? What has the funis at all to do with the matter? The author very properly observes, however, that—

"We must come out of our retreat and mingle with the busy hum of men, rub off our rust by friendly collision, and submit ourselves to the rasp of irony and criticism, in order to lower our pointed and consequential conceits." 44.

4. *Aphonia.* We will do Mr. Sutleffe the credit of being a most candid narrator of his experience. He relates the unsuccessful as well as the successful events of his practice—which is no mean merit in these days. Our author is a great admirer of old women—and observes, page 55, "that old women do as well as old men—and sometimes better." In illustration of this aphorism, he mentions two cases of aphonia which completely baffled him. The patients applied to an old woman in the neighbourhood, who speedily cured them with "gin and oatmeal."

5. *Fatal Hæmorrhage from a Bubo.* Mr. Sutleffe was summoned to a young man with sloughing bubo, and where "the pulsation of the iliac artery was awful." The artery burst and the patient quickly bled to death. "I have since thought that a ligature applied to the artery might have suspended the bleeding, if not saved his life." On returning home, our author reflected on those apocryphal words—"Oh, Adam! what hast thou done?" We would beg leave to add—Oh, Sutleffe! what hast thou *left undone?*

6. *On Flannel.* Mr. Sutleffe has a mortal antipathy to flannel—and why? Because it is prohibited by the Prophet Ezekiel, [xliv.] in these words—"They shall not gird themselves with wool that causeth sweat." In corroboration of scripture he has observed, "that those who continue flannel in immediate contact with the skin, are more susceptible of catarrh or quinsy than others." He has long noticed this fact—but admits that "he may be under a delusion."

7. *Uric Acid.* Mr. Sutleffe is possessed of an immense number of nostrums, picked up in the thirty-eight years experience. One is a compound of rhubarb, soap, and oil of juniper which *sweeps out* the kidneys, ureters, and bladder, when incommoded with red gravel. "I call it the *besom,* and it is thought to be an appropriate name in the circle in which I move." 167.

8. *Medical Etiquette.* Mr. Sutleffe has favoured the profession with a short sermon on this interesting subject, the nucleus or text of which appears to be this. He went on an excursion to the North, and entrusted his business, pro tempore, to a neighbouring practitioner, who was a German, and who, with shame be it told, diddled Mr. Sutleffe out of a patient.—Mr. S. meeting the German one day afterwards, accosted him thus:—"You should have been acquainted with the Dutch Proverb, that—*there is nothing got by stealing, nor lost by praying.*" Mr. Sutleffe's prayers appear to have prevailed; for some time afterwards the German "was literally kicked out of doors" by the patient whose allegiance he had perverted.

9. *Horehound Tea keeps a Saint out of Heaven upwards of Twenty-four Years.*

"In the autumn of 1800, I was asked if I wished to see a triumphant saint expire. 'Much more,' I replied, 'than to see Rome in all her pristine or present glory.' I was accordingly directed to call on Mrs. W——, of the Surrey-road, which I did, in whom I beheld the nearest approach to an animated skeleton I ever expected to see. She instantly recognised me (having often met me at the sanctuary,) and shook hands feebly. She was 'on the mount of God's unchangeing love.'" 242.

Mr. Sutleffe had the cruelty to administer horehound tea, which prevented the saint from "going home." When informed that she was out of danger, she shed tears of grief. She has retired to Warwickshire instead of Paradise, and is got quite lusty. What a misfortune!

10. *Sleep.* Mr. Sutleffe can give you a scriptural recipe for every thing. It is often asked, and foolishly asked, how many hours should we sleep? Mr. Sutleffe appears to like a comfortable nap, and quotes the Bible, as usual, for the best authority. "It is vain for you to sit up late, to rise early, and eat the bread of sorrows."—*Psalms.* John Wesley ("whose reward in Heaven I presume is *far superior to that assigned to the Rev. John Toplady*") adjudged six hours sleep to a man, seven to a woman, and eight to a fool. Mr. Sutleffe's "chosen minister often smiles and says we all require the fool's hours." 281.

11. *Breath of a Child sucked by a Cat.* Mr. Sutleffe has had "*proof positive*" of this fact, (though he saw nothing of the circumstance) in a case, where the father actually caught puss stealing the breath from his infant in the cradle! It was fourteen days before the child recovered from the effects of this wicked theft of the cat.

12. *Snuff.* Mr. Sutleffe takes snuff *only* on two occasions. 1st. "To prevent a dozing disposition at public worship"— Oh, fie, St. Sutleffe! And 2ndly. "To correct a repulsive exhalation from a diseased subject." He lately got the contagion of scarlatina in his nose, having left his snuff-box behind him. A pinch of snuff, procured at Stone's End, Borough, completely destroyed "all sense and idea of contagion."

13. *Presence of Mind in a Resurrection-man.* A mendicant on London Bridge, while bolting a piece of meat in a hurry, was suffocated on the spot by a portion entering the glottis. A crowd was quickly collected. A resurrection-man rushed through them, burst into tears, and cried, oh, my brother! The sympathising spectators soon procured a hackney-coach, in which the resurrection-man quietly conveyed the corpse to a dissecting room, and pocketed the premium.

By this time our readers will begin to think they have had specimens enough of the volume under review, and so think we. About 100 pages of the conclusion of the work are occupied with miscellaneous papers, not medical but chiefly religious. Of these we shall only present our readers one single sample. *Mr. Sutleffe* having been an eye witness of the transaction, it must of course be credited, as of recent occurrence; but, if our memories do not deceive us, there is some thing of the kind in Joe Miller.

"During my attendance upon Mrs. M——, of Creed-lane, one Monday morning, to take my leave *pro formâ*, she astonished me with the account of the following occurrence:—' O! Sir, I wish you had been here yesterday morning, the whole neighbourhood was in a roar of laughter: that house,' pointing to the south, ' has long borne a bad character. On Saturday evening an old gentleman brought in a young lass,' (the latter, by the way, was the lesser delinquent), ' and early on the following morning she contrived to escape without the knowledge of her paramour, and took with her his large bundle, exulting in her unknown prize, and exclaiming she had ' bilked the old fellow.' And what do you think it contained? Why nothing less than the sacerdotal robes of a dignified clergyman. The poor man, upon the discovery of this loss, could not refrain from uttering his grief and disappointment before them all; for ' what shall I do?' he observed; she had even taken his sermon with her, and he had engaged to preach it for a public charity, a few miles north west of the metropolis, (I am unwilling to exhibit the name of the place,) where he told his wife and daughters he should sleep.' 'O! Sir,' added Mrs. M——, 'what a crying shame!'

"It was not possible, for some period after the recital, to compose myself so as to *appear sober abroad*; and I was obliged to wait until I could get my risible muscles into some order. On my reaching home, we had a second edition of the story." 577.

What an edifying homily for Mrs. Sutleffe and her daughters! In taking leave of our author we will give him a piece of advice. We recommend him to distribute the whole impression of his work among his patients and friends. The numerous, or rather innumerable cases it contains will do just as well for them as the best specimens of pathology and practice that ever saw the light. The work will impress them with a vast idea of Mr Sutleffe's skill, experience—and godliness. It will thus make his fortune; whereas among the medical profession it never will have any circulation—and if it had, it would only injure the author. This is rough, but it is wholesome advice.

